# Revolutionizing Food Safety with Quantum Dot–Polymer Nanocomposites: From Monitoring to Sensing Applications

**DOI:** 10.3390/foods12112195

**Published:** 2023-05-30

**Authors:** Tushar Kanti Das, Sayan Ganguly

**Affiliations:** 1 Institute of Physics–Center for Science and Education, Silesian University of Technology, Krasińskiego 8, 40-019 Katowice, Poland; 2 Bar-Ilan Institute for Nanotechnology and Advanced Materials, Ramat Gan 5290002, Israel

**Keywords:** quantum dots, polymer, nanocomposites, toxicity, microorganisms, monitoring

## Abstract

The present review article investigates the prospective utilisation of quantum dot–polymer nanocomposites in the context of ensuring food safety. The text pertains to the advancement of nanocomposites, encompassing their distinctive optical and electrical characteristics, and their prospective to transform the detection and perception of food safety risks. The article explores diverse methodologies for producing nanocomposites and underscores their potential utility in identifying impurities, microorganisms, and harmful substances in food. The article provides an overview of the challenges and limitations associated with the utilisation of nanocomposites in food safety applications, encompassing concerns regarding toxicity and the necessity for standardised protocols. The review article presents a comprehensive examination of the present research status in this area and underscores the potential of quantum dots–polymer nanocomposites in transforming food safety monitoring and sensing.

## 1. Introduction and Background

Nanosized semiconductor particles known as quantum dots (QDs) display novel quantum mechanical features, including optical, electrical, and magnetic capabilities that vary with QD size [1,2]. Because of these characteristics, they are of great interest for use in fields as diverse as electronics, photonics, energy harvesting, and biology. While polymers are big molecules composed of repeating units, polymer nanocomposites mix polymers with nanoscale fillers such as quantum dots to generate materials with improved characteristics and functionalities [3,4]. Nanotechnology, which studies and makes use of matter at the nanoscale, is the cradle of the quantum dot concept (typically below 10 nm) [4]. QDs have special features because of the quantum confinement effect; they are commonly made of inorganic semiconductors such as cadmium selenide (CdSe) [5,6], lead sulphide (PbS) [7,8], or indium phosphide (InP) [9,10,11]. When the material’s dimensions are shrunk to an order of magnitude less than the de Broglie wavelength of the electrons, quantum mechanical phenomena manifest [12]. This means that QDs have features that change as their sizes do, such as high quantum yields and a broad absorption spectrum that can be tuned to emit light in a variety of colours [13,14]. On the other hand, polymer nanocomposites are types of high-tech materials that incorporate nanoscale fillers into polymers to make products with specific characteristics [15]. Because of their adaptability, processability, and vast variety of characteristics, polymer matrices are frequently utilised as host materials. Improvements in electrical conductivity, mechanical strength, and even optical qualities can result from incorporating quantum dots as nanoscale fillers into polymer matrices [16]. By controlling the quantum dot’s size, shape, and concentration in addition to the polymer matrix, the properties of the resulting polymer nanocomposites can be finely adjusted. Nanocomposites, which include quantum dots and polymer matrices, have a wide variety of possible uses. Because of their exceptional optical properties, QD–polymer nanocomposites have been investigated for use in optoelectronic devices such as LEDs, solar cells, and sensors [17]. Because of their superior mechanical qualities and processability compared to pure quantum dot materials, QD–polymer nanocomposites have found applications in flexible electronics, wearable devices, and displays. Many other fields stand to benefit from the widespread use of these materials in the future, from electronics and photonics to biology and energy harvesting. Quantum dot and polymer nanocomposite research is a promising new area of materials science and nanotechnology because of their unusual characteristics and wide range of possible applications [18,19].

Applications in the food sector rely heavily on monitoring and sensing technologies to ensure that food is prepared and served safely [20]. These procedures are critical for guaranteeing that the food we eat is healthy, untainted, and of the highest standard. First and foremost, ensuring that food is free from hazardous germs such as bacteria, viruses, and parasites is crucial in preventing customers from becoming unwell [21]. They can result in a wide variety of health problems, from moderate gastrointestinal distress to life-threatening infections that necessitate hospitalisation, or even death. Quantum dot (QD)–polymer composites are finding increasing use in a wide variety of food applications, and protecting the integrity of these products is of the utmost significance. In the food industry, QD–polymer composites are widely used for food packaging. These composites are used to make modern packaging films that delay deterioration, cut down on oxidation, and thwart the growth of hazardous microbes in food [22,23,24]. However, to make sure that no dangerous compounds from the composite seep into the food and endanger consumers’ health, it is crucial to monitor the safety of these materials. Using QD–polymer composites as sensors for identifying food pollutants also requires careful monitoring of food safety. In order to identify contaminants such as pesticides, heavy metals, and pathogens in food samples, these composites can be engineered to behave as highly sensitive and selective sensors [25]. Consistent sensing and monitoring of these composites is essential for ensuring the accurate detection of pollutants and preventing unsafe food from reaching the market. In addition, QD–polymer composites for food quality control rely heavily on safety and monitoring measures [14,26]. Indicators of freshness, maturity, and spoiling can be derived from these composites. Producers and processors may ensure their products are up to code and safe to eat by keeping an eye on these metrics [27]. The food business and customers would both benefit from the consistent monitoring and testing of these composites to guarantee their safety and efficacy in maintaining food quality, identifying pollutants, and enhancing packaging materials [28]. Maintaining the quality of food products and safeguarding the health of consumers depend on the reliability of the QD–polymer composites used in their production [29]. Therefore, it is crucial to put food safety and monitoring at the forefront of QD–polymer composite research, development, and use in the food sector. Protecting the health of consumers and keeping food products intact necessitates strict regulations for the usage of QD–polymer composites in the food industry [30]. In this review, we will discuss the fabrication and properties of QD–polymer hybrids and their mode of sensing/monitoring applicability towards foods industry/science.

## 2. Methods for Synthesis of QD–Polymer Nanocomposites

Composite materials consist of two distinct phases, namely, a continuous matrix and a discontinuous filler or reinforcement. In the context of nanocomposites, the reinforcing material possesses a dimension that measures less than 100 nm, specifically in the nanometre scale [31]. The functional role of enhancing the attributes of the final material for particular uses is determined by the filler present in the nanoscale size [20]. In the realm of QD–polymer nanocomposites, the matrix material may consist of either a linear or branched thermoplastic or a thermoset (crosslinked polymer) [32,33]. The preparation method used can determine whether the polymer matrix will be mixed non-covalently or covalently grafted with the quantum dot filler. The selection of the polymer material is contingent upon its intended use within a particular domain. The primary role of the matrix in polymer composites is to facilitate the binding of the QD filler and to impart the necessary strength characteristics, which are contingent upon the chemical composition of the thermoplastic or thermoset polymer [34]. The potential for QD dispersion within the matrix is contingent upon the capacity to attach the QD, which is, in turn, determined by the chemical composition of the polymer. Various methods exist for the preparation of QD–polymer composites, including chemical, physical, and in situ growth approaches.

## 3. Synthesis Approaches of Physical Mixing

**Figure 1:**
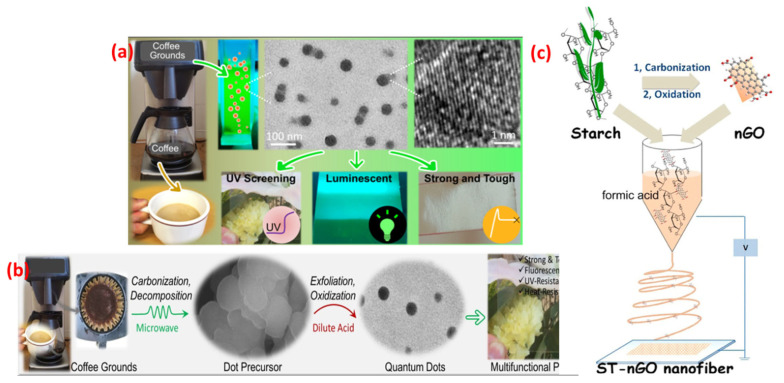
(**a**) Fabrication method of QDs and multifunctional biocomposites (**b**) Schematic illustration of the structural transformation from used coffee grounds to dot precursor and homogeneous incorporation of QDs into PLLA for multifunctional biocomposites [42]. (**c**) Diagrammatic overview of the electrospinning process for the production of ST-nGO nanofibers. Reproduced with permission from Ref. [43]. © 2017 American Chemical Society.

The simplest procedure to synthesise a QD–polymer composite requires physically combining the two components (QD and polymer). This can be achieved by incorporating a small amount of QDs into the polymer and then casting the resulting mixture. In this instance, non-covalent interactions including electrostatic interaction, hydrogen bonding, or the interaction between the QDs and the polymer lead to the formation of the nanocomposite [4,35,36,37,38]. The electrostatic interaction is commonly regarded as a weaker bond in comparison to ionic or covalent bonding, yet it is stronger than the van der Waals interaction that occurs between non-polar molecules [20]. The primary mechanism of hydrogen bonding is through electrostatic interaction. Hydrogen bonding occurs when a hydrogen atom that is partially positively charged in one molecule interacts with a nearby molecule that contains a negatively charged atom, specifically fluorine (F), nitrogen (N), or oxygen (O). Electrostatic interactions may be associated with either attractive or repulsive forces between molecules that are charged. The physical mixing technique employed in QD–polymer composites is primarily governed by attractive interactions, which serve as the primary driving forces for achieving homogeneous mixing and the consequent enhancement of material properties [38]. The negative charge observed in QDs can be attributed to the interaction between functional groups, such as carboxylic groups, and the positive charge of polymers [39]. QDs typically display negative surface charges owing to the existence of carboxylic functional groups, thereby leading to interactions with polymers possessing a positive charge [40]. The preparation of a carbon quantum dot–polymer nanocomposite was carried out by Xu et al. using the physical mixing technique [41]. The present investigation involved the integration of GO-QDs with high oxygen content, derived from coffee grounds, into bionanocomposite films of polylactide acid (PLA). This integration facilitated the development of advantageous interfacial interactions, which, in turn, aided the exfoliation and even distribution of nGO-QDs within the matrix. Another group of researchers reported the utilisation of a microwave-assisted technique that facilitated the production of luminescent QDs that have an affinity for polymers on a gram-scale using used coffee grounds. The zero-dimensional QDs were endowed with appropriate exfoliation and uniform dispersion in a poly(l-lactic acid) (PLLA) matrix (Figure 1a,b) due to their ultrasmall dimensions (~20 nm), and the presence of a variety of oxygen functional groups [42]. Composite electrodes comprising PPy/CQDs were produced on a graphite sheet using a controllable galvanostatic electropolymerisation technique by Malik et al. [37]. The aforementioned procedure facilitates the regulation of polymer thickness, whereas the current density exerts an influence on the film’s morphology and conductivity. The composite material composed of PPy/CQDs exhibited exceptional electrochemical performance, displaying a noteworthy specific capacitance in comparison to pure PPy. This suggests that the composite material holds promise for utilisation in energy storage devices. In another work, Wu et al. employed starch-derived nGO-QDs (Figure 1c) as property modifiers to fabricate electrospun nanofibrous scaffolds [43]. The incorporation of nGO-QDs has exhibited a significant enhancement in the electrospinnability of starch, thereby leading to an improvement in the thermal stability of the resultant spun fibres. QDs, particularly those based on graphene, possess a high concentration of conjugated sp^2^ domains. The graphene-based quantum dots exhibit a high propensity to engage in π–π interactions with polymers that contain aromatic groups, such as polypyrrole and polystyrene, or with polymers that are functionalised with p-orbital-rich groups, such as pyrene and phenyl [37]. The physical mixing methodology offers several benefits, including ease of operation, cost-effectiveness, and scalability for industrial applications, particularly in the context of melt processing. However, the phenomenon of agglomeration poses a challenge due to the self-π–π stacking interactions between QDs and polymer, which can hinder the homogeneous dispersion of QDs in the polymer matrix. This can result in poor mechanical performance and unstable optical characteristics.

**Figure 2:**
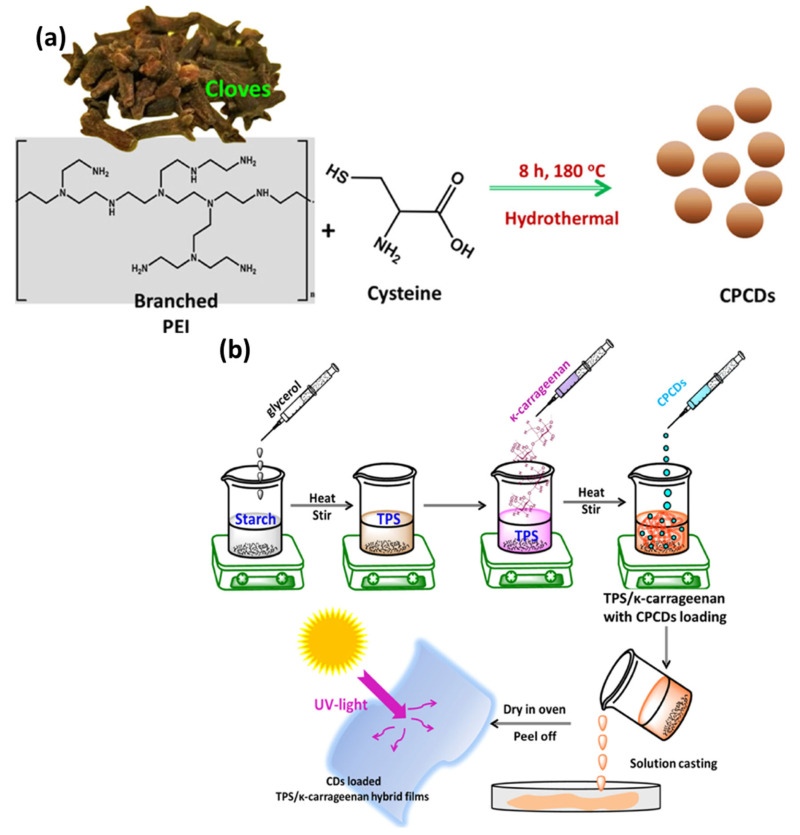
(**a**) Schematic illustration for the preparation of CQDs through a hydrothermal approach. (**b**) Graphical representation pertaining to the preparation method of a TPS–κ-carrageenan film loaded with CQDs. Reproduced with permission from Ref. [4]. © 2022 American Chemical Society.

In a report, Das et al. employed a physical compounding method to achieve functional films based on biopolymers that are reinforced with heteroatom-doped CQDs [4]. The process of synthesis and fabrication can be defined into two discrete stages. The first stage involved the preparation of nitrogen- and sulphur-doped CDs through a facile hydrothermal method, as shown in Figure 2a. The subsequent phase involved the production of hybrid thin films that incorporated CDs through the employment of the single-step ‘cast and peel’ technique (Figure 2b). The polymeric hybrid films of TPS and κ-carrageenan loaded with CQDs exhibited remarkable antioxidant activity for both the DPPH assay (over 85%) and ABTS assay (over 90%). Furthermore, the polymeric films loaded with CDs exhibited antimicrobial properties against model strains of both Gram-negative *E. coli* and Gram-positive *B. subtilis*. The film composed of TPS–κ-carrageenan and reinforced with CQDs exhibited favourable optical, mechanical, UV-protective, and antioxidative characteristics, thereby potentially extending the application in the storage duration of food products.

## 4. Synthesis Approaches of In Situ Polymerisation

While the physical mixing method is a straightforward approach for the preparation of QD–polymer composites, it is characterised by a weak intermolecular interaction between the QDs and the polymer matrix. The utilisation of in situ chemical grafting techniques facilitates the establishment of a robust interfacial connection between QDs and the polymer matrix through the creation of potent covalent bonds. The process of in situ growth is known to be both time and energy intensive, as it involves multiple steps, complex reaction processes, and the use of toxic chemical reagents and solvents. In contrast to alternative methods, the in situ synthesis of quantum dots within the polymer matrix is deemed more favourable due to the chemical and physical interactions that occur between the QDs and the polymer, resulting in improved attachment. The in situ synthesis methodology may be accomplished through a one-pot thermal treatment technique involving either pyrolysis or the low-temperature heating of a mixture comprising QD precursors and a monomer, or via a hydrothermal process [44,45]. The simultaneous formation of QDs and the polymer precursor matrix occurs in a single reaction vessel. The immobilisation of QDs within a matrix presents certain drawbacks, including limited compatibility between the two constituents, resulting in anisotropic characteristics. The QDs will initiate and develop on the active sites of the polymer matrix, resulting in a system that is both highly stable and homogeneous [46]. Upon completion of the synthesis process, it is possible to cast the solution of QD–polymer composites into a film or sheet. The manipulation of the polymer structure enables the modulation of QD size and uniform distribution within the polymer matrix. The effective dispersion of QDs within a matrix can be achieved through the utilisation of either sonication or a suitable surfactant.

## 5. Chemical Grafting Method

**Figure 3:**
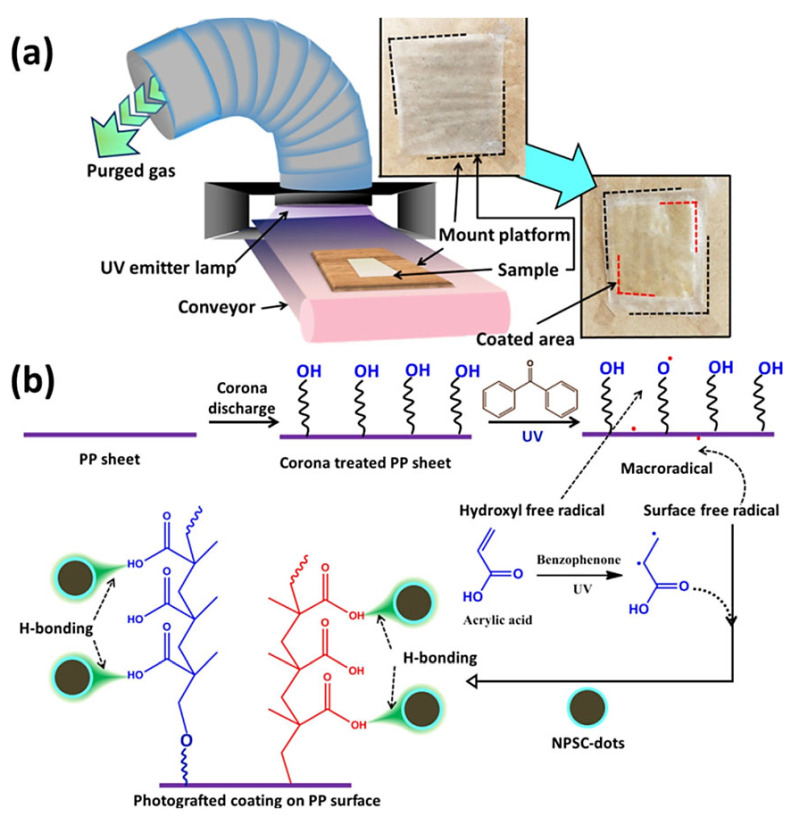
(**a**) Schematic illustration of the coating procedure (inset shows the uncoated and coated films). (**b**) Plausible grafting reaction mechanism between polypropylene and acrylic acid. Reproduced with permission from Ref. [50]. © 2021 American Chemical Society.

The surface of quantum dots exhibits an enormous functional group, thereby increasing the potential for chemical modifications that can lead to the formation of a covalent network with the polymer. Various chemical reactions such as esterification [47], methacrylation/acrylation, acylation, etherification, and epoxidation have been observed [48]. The chemical grafting method is considered superior to the physical mixing technique due to its ability to provide uniform mixing, mechanical strength, and an extended life cycle. This is attributed to the generation of covalent bonds between the QDs and the polymer chain. In their study, Gustavsson et al. synthesised a thermoplastic composite film composed entirely of cellulose through the covalent functionalisation of cellulose acetate using oxidised carbonised cellulose/cellulose-based oxidised carbonised cellulose (OCC) [49]. The OCC was prepared through a microwave-assisted hydrothermal carbonisation process of paper tissues and cellulose. The OCC was produced using a technique known as the microwave-assisted hydrothermal carbonisation (HTC) of cellulose, which was subsequently followed by oxidation and dialysis. The micrometre-sized OCC exhibited a planar morphology and was found to possess diverse oxygen functionalities, which facilitated its conversion into acyl-chlorinated OCC through the application of moderate reaction conditions. The study involved the production of composites of OCC-modified cellulose acetate (CA) and neat CA, which were synthesised using the recyclable ionic liquid 1-allyl-3-methylimidazolium chloride. The study aimed to investigate the impact of varying the degree of acetylation and amount of OCC on the thermal and physical properties of the composites. The composites of CA modified by OCC exhibited a significantly improved capacity for film formation, resulting in enhanced optical and mechanical characteristics in comparison to pure CA. Furthermore, the research demonstrated the feasibility of producing OCC-modified CA composites utilising waste materials, specifically paper tissues. The utilisation of OCC modification has been shown to hold significant potential in the development of robust and transparent thermoplastic composites composed entirely of cellulose, while still maintaining a reasonable degree of flexibility. In their study, Das et al. proposed a novel fluorescent coating that was successfully synthesised on a polypropylene film using CQDs via the photografting technique (Figure 3) [50]. This represents the first instance of such a coating being developed. Prior to the application of fluorescence coating using C-dots, the polypropylene film underwent corona treatment, which involved subjecting it to high voltage (21–28 kV) to facilitate the introduction of functional groups on its surface. Poly(acrylic acid) (PAA) was covalently grafted onto a polypropylene film surface through photografting reactions after surface treatment. A hybrid coating was fabricated using a grafting mixture of acrylic acid and C-dot solution. Polypropylene (PP) films were modified by grafting acrylic acid onto their surface through the formation of a poly(acrylic acid) coating, which was subsequently physisorbed over CQDs. CQDs have the ability to serve as anchors for polymers through physical interactions such as H-bonding or other mechanisms.

## 6. Food Safety Applications of QD–Polymer Nanocomposites

QD–polymer nanocomposites can improve food packaging, detect and monitor foodborne pathogens, and assess food quality using sensing and imaging. These nanocomposites detect pollutants in food quickly and sensitively, improving food safety and consumer protection [51].

Polymer nanocomposites with quantum dots (QDs) improve packaging monitoring, and the key reasons to consider them are outlined below.

Quantum dots’ optical properties can be used for sensing. Size-dependent fluorescence emission allows for the sensitive and tuneable detection of physical and chemical parameters. Quantum dots in polymer nanocomposites can create a highly responsive sensor for packaging conditions such as temperature, humidity, oxygen levels, and chemical contaminants. Polymer nanocomposites with quantum dots enable real-time packaging monitoring. Fluorescence spectroscopy is a simple, non-destructive method for measuring quantum dots’ optical properties. This allows continuous packaging condition monitoring, providing valuable information on the integrity, freshness, and quality of packaged contents throughout the supply chain. Quantum dots can monitor many packaging parameters due to their wide sensing range. Quantum dot–polymer nanocomposites can respond to stimuli by emitting different fluorescence wavelengths. Multiplexed sensing can be caried out using different quantum dot–polymer combinations in the same packaging material. In terms of durability and stability, polymer nanocomposites protect quantum dots. Oxygen, moisture, and mechanical stress can degrade quantum dots, but the polymer matrix protects them. This ensures long-term sensing functionality and reliability, allowing prolonged packaging condition monitoring without significant degradation or loss of sensitivity. They are also cost-effective, as scalable colloidal synthesis can create quantum dots cheaply. Solution blending or melt processing can create polymer nanocomposites cheaply. Quantum dot–polymer nanocomposites are cost-effective for large-scale packaging monitoring due to the low cost of quantum dots and scalable polymer processing methods.

QD–polymer nanocomposites can also improve food packaging, monitoring, and quality control [52]. These nanocomposites could improve food safety and quality. However, more study and development are needed to fully grasp their food industry safety and regulatory and commercial feasibility [53]. To ensure food safety, nanomaterials must be properly assessed and managed. QD–polymer nanocomposites in food safety could help the food industry, consumers, and public health [54]. QD–polymer nanocomposites are predicted to improve food safety and quality as research advances. Like with any new technology, regulation and oversight are essential for food sector safety. The overuse of pesticides has raised concerns regarding food safety. The advancement of expeditious and precise detection methodologies for these chemical entities is an expanding area of investigation. The present study reports the successful synthesis of fluorescent nanocomposites comprising cadmium sulphide quantum dots (CdS-QDs) and polymer matrices of chitosan, carboxymethylcellulose, and pectin [55]. As per documented reports, a significant number of individuals worldwide, exceeding 200 million, are subjected to levels of arsenic (As) contamination that surpass the World Health Organization’s (WHO) recommended threshold of 10 μg/L [56]. The present study describes the development of a newly designed aptasensor with fluorescence OFF-ON capabilities, which exhibits high sensitivity for the detection of As (III). In order to fabricate the fluorescence aptasensor, novel AuFeZnSe-alloyed quantum dots (QDs) were synthesised and coated with amphiphilic polymers (Amp-P) on their surface to confer biocompatibility and stability to the QDs [57]. Subsequently, the process involved the electrostatic bonding of cationic gold nanorods (AuNRs) to the Amp-P-QDs, resulting in the formation of an Amp-P-QDs-AuNR nanocomposite. This composite effectively suppressed the fluorescence of the bound QDs. Antimicrobial properties can be incorporated into QD–polymer nanocomposites through engineering. The integration of antimicrobial agents, such as silver nanoparticles or antimicrobial peptides, into the polymer matrix of nanocomposites has been shown to be an effective strategy for preventing the proliferation of bacteria and other microorganisms on food surfaces [58]. The implementation of this measure can potentially mitigate the likelihood of foodborne illnesses and prolong the longevity of perishable commodities [24]. Polymer nanocomposites containing quantum dots (QDs) can be customised for the purpose of detecting allergens present in food [59]. The process of functionalising quantum dots (QDs) with allergen-specific antibodies or aptamers enables their selective binding to allergenic proteins, thereby eliciting a quantifiable response [60]. The utilisation of this technology has the potential to facilitate the expeditious and precise identification of allergenic substances, including those present in minute quantities, thereby mitigating the risk of allergic responses and safeguarding the well-being of individuals afflicted with food allergies [61].

## 7. Detection and Monitoring of Foodborne Pathogens Using QD–Polymer Nanocomposites

**Figure 4:**
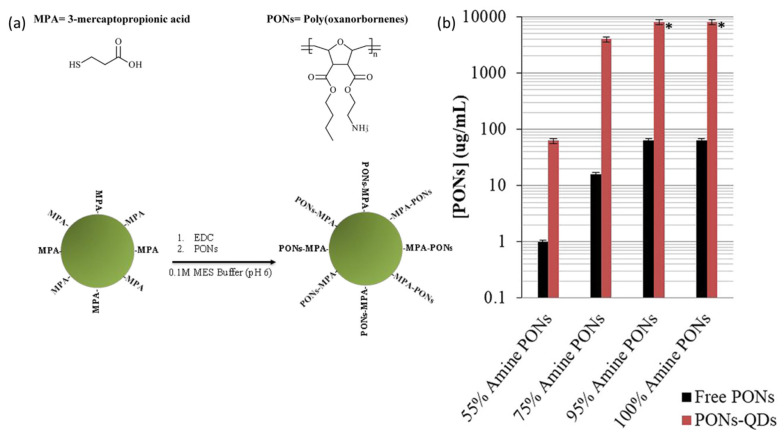
(**a**) Synthesis of CdTe quantum dots (QDs) coated with poly(oxanorbornene) (PONs) for potential applications in various fields. (**b**) PONs-QD HC50 values vs. free PONs molecules. PONs-QDs (red) had reduced haemolytic activity than free PONs (black) after red blood cell exposures to 0.1–8000 μg/mL PON equivalents (three biological replicates). *: HC50 values for 95% and 100% Amine PONs-CdTe QDs were not found within the tested concentration range, and are thus represented as bars to the highest tested concentration. Reproduced with permission from Ref. [63]. © 2020 American Chemical Society.

The occurrence of pathogenic bacteria in food can result in foodborne illnesses that can have grave health implications, including fatality. The identification and surveillance of foodborne pathogens are essential for guaranteeing the safety and excellence of food commodities [62]. The utilisation of quantum dot–polymer nanocomposites in food packaging applications is a potentially effective method to attain this objective. Foodborne microorganisms pose a significant public health risk on a global scale. According to the World Health Organization (WHO), an estimated 600 million individuals become ill and 420,000 individuals perish each year as a result of foodborne illnesses caused by pathogens such as salmonella, campylobacter, listeria, and *Escherichia coli* (*E. coli*) [60]. Foodborne pathogens have the potential to taint food at various points along the food supply chain, such as during production, processing, packaging, and transportation. Conventional techniques utilised for the identification of foodborne pathogens entail laborious and expensive laboratory examinations. The aforementioned techniques frequently necessitate multiple days to procure outcomes and may demand significant physical effort. Moreover, none of the above techniques can offer real-time monitoring, which makes it hard to guarantee the safety and quality of food throughout the whole supply chain. Nanotechnology has surfaced as a viable method for the identification and surveillance of foodborne pathogens [52]. A study was conducted wherein CdTe quantum dots (QDs) (Figure 4a) were coated with synthetic mimics of antimicrobial peptides derived from poly(oxanorbornene) molecules (PONs) [63]. The study examined the efficacy of PONs-CdTe QDs against *Escherichia coli* (Figure 4b), a bacterial strain known for its resistance to antibiotics. Simultaneously, a comparison was made between the antibacterial efficacy of PONs-CdTe QDs and that of unbound PONs and CdTe QDs. The antibacterial activity of PONs-CdTe QDs was found to be both concentration dependent and additive in nature. The results of the study indicate that the conjugates exhibited a notably reduced minimum inhibitory concentration (MIC) in comparison to the unbound PONs and QDs. This was particularly evident in the case of PONs-CdTe QDs, which were composed of PONs with a high amine density. The optimal performance of PONs-CdTe QDs was not achieved through the attachment of PONs with the greatest inherent antibacterial activity (i.e., the least MIC in solution as unbound PONs), suggesting that the mode of operation for unbound PONs and PONs-CdTe QDs is distinct.

Nanotechnology pertains to the manipulation of matter at the nanoscale, which corresponds to the scale of atoms and molecules. The development of nanocomposites for food packaging is a promising application of nanotechnology in the realm of food safety. Nanocomposites refer to substances comprising a polymer matrix that incorporates nanoscale fillers, such as QDs. QDs are semiconductor nanoparticles that exhibit fluorescence properties, emitting stable and intense fluorescence signals upon excitation by light. The fluorescence emissions exhibited by quantum dots (QDs) are known to exhibit a high degree of sensitivity to alterations in their immediate surroundings, including, but not limited to, variations in pH levels, temperature fluctuations, and the existence of particular molecules. The integration of quantum dots and polymer nanocomposites in food packaging materials presents a novel strategy for the identification and surveillance of foodborne pathogens [64]. Nanocomposites have the potential to exhibit pathogen-specific interactions, thereby eliciting a fluorescence modulation upon pathogen detection. The alteration in fluorescence can be identified through the utilisation of specialised equipment, thereby furnishing instantaneous data regarding the existence of pathogens in comestible items [65]. When it comes to food packaging, using nanocomposites made of quantum dots (QDs) and polymers has many advantages over traditional methods of detection. The aforementioned benefits encompass elevated levels of sensitivity, specificity, and expeditiousness. Quantum dots (QDs) have exceptional photostability; thus, their fluorescence signals last for a long time, making them suitable for long-term monitoring. Furthermore, the utilisation of nanocomposites comprising quantum dots (QDs) and polymers can offer a means of monitoring food products that is non-invasive and non-destructive, thereby mitigating the requirement for costly and protracted laboratory analysis. Notwithstanding the benefits, the utilisation of QD–polymer nanocomposites in food packaging applications presents certain difficulties. A significant obstacle that arises in the utilisation of quantum dots (QDs) is the plausible occurrence of toxicity. CdSe and PbS QDs contain toxic heavy metals. If released from quantum dots and accumulated in organisms or ecosystems, these heavy metals can harm humans and the environment [66]. Quantum dots can cause cellular and genotoxicity. Quantum dots release heavy metal ions, which can cause oxidative stress and DNA damage. These effects may cause biological responses and health issues [67]. Researchers and industry stakeholders are addressing quantum dot toxicity. Quantum dots made of indium or zinc instead of cadmium or lead are being developed. To reduce heavy metal release and improve quantum dot stability and biocompatibility, surface modifications and encapsulation are being investigated [68]. Several research studies have indicated that specific varieties of quantum dots (QDs) may possess cytotoxic properties towards cells and living organisms [30]. Adegoke et al. have documented the development of a new fluorescence aptasensor for detecting As (III) through the utilisation of a hybrid nanocomposite consisting of non-cadmium-containing amphiphilic polymer (Amp-P)-coated AuFeZnSe alloyed QDs and gold nanorods (AuNRs) [69]. In this study, novel AuFeZnSe alloyed quantum dots (QDs) were synthesised and utilised as the selected fluorophore signal reporter. This choice was made in lieu of the frequently employed cadmium-based Group II-VII fluorescent QDs, which have been associated with potential safety risks.

## 8. Food Spoilage Detection Using QD–Polymer Nanocomposites

The application of quantum dot (QD)–polymer nanocomposites for the detection of food spoilage is an area of research that has garnered considerable attention in recent times. Quantum dots (QDs) are semiconductor nanoparticles that exhibit light emission upon excitation by an external stimulus. The different types of possible detection methods adopted by commercial sectors are tabulated in Table 1. Due to their distinctive optical and electronic characteristics, QDs have found utility in a range of applications, including sensing. Nanocomposites comprising quantum dots (QDs) embedded within a polymer matrix have the potential to detect a range of gases, including those that are typically released during food spoilage. When gas molecules come into contact with the quantum dots (QDs) in the nanocomposite, the nanocomposite sends out a clear signal that can be used to detect gases. By watching how the signal changes over time, you can determine how much food has spoiled.

**Figure 5:**
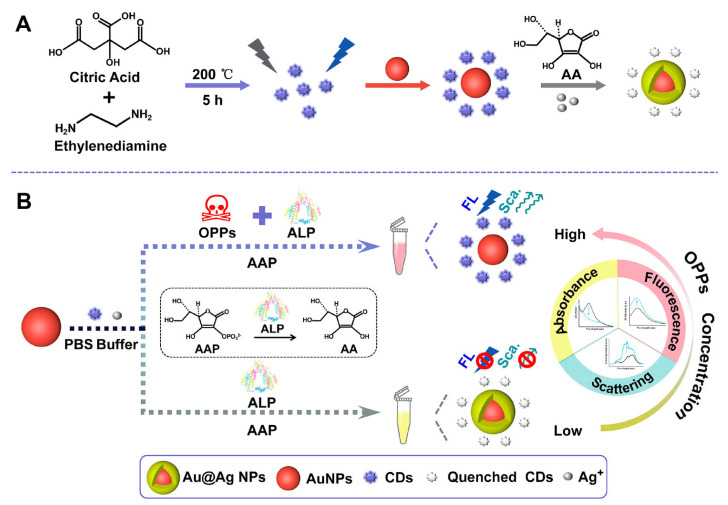
Schematic illustrations depicting (**A**) the synthesis of cyclodextrins (CDs) and the subsequent formation of a gold–silver (Au@Ag) nanostructure, as well as (**B**) the triple-signal sensing platform utilised for the assay of organophosphorus pesticides (OPPs). Reproduced with permission from Ref. [80]. © 2022 American Chemical Society.

The utilisation of plasmonic nanoparticles holds significant potential in the development of sophisticated optical and electrical sensors. Liu et al. introduced a multifaceted sensing approach capable of the triple-mode detection of organophosphorus pesticides (OPPs) utilising carbon dots (CDs) (Figure 5) in conjunction with silver deposition on gold nanoparticles (AuNPs). A hybrid system comprising AuNPs, CDs, and silver (Ag) ions in phosphate-buffered saline (PBS) has been formulated [80]. The development of an Au@Ag nanostructure results in concomitant alterations in absorption, fluorescence, and Rayleigh scattering. The enzyme inhibitor OPPs have been found to effectively inhibit the activity of ALP. As a result, a sensing system that utilises triple-signal enzyme inhibition has been developed for the detection of OPPs. The integration of a nanohybrid sensing platform with a triple-signal readout system has yielded novel findings that could advance the field of optical sensors.

## 9. Detection of Chemical Contaminants in Food Using QD–Polymer Nanocomposites

The optical and electronic properties of quantum dot (QD)–polymer nanocomposites render them a promising material for detecting chemical contaminants in food [81]. Quantum dots (QDs) are a type of semiconductor nanocrystals that exhibit light emission upon excitation by a light source [82,83]. The emission wavelength of QDs can be modulated by manipulating their size and composition [84]. Polymer nanocomposites are composite materials comprising a polymer matrix and dispersed nanoscale particles. The amalgamation of quantum dots (QDs) with a polymer matrix has the potential to yield a composite material that exhibits superior characteristics, including heightened sensitivity and selectivity [85,86,87]. In the process of identifying chemical contaminants in food through the utilisation of QD–polymer nanocomposites [88,89], it is customary to functionalise the QDs with a distinct ligand that exhibits selective binding properties towards the intended contaminant. Upon exposure to the contaminant, the quantum dots (QDs) undergo a binding event with the ligand, resulting in a discernible alteration in either the fluorescence intensity or wavelength of the QDs [90]. This change can be detected through the use of a fluorescence spectrometer. QD–polymer nanocomposites offer a rapid and sensitive detection method for chemical contaminants in food, which is considered an advantage [91]. Furthermore, they possess the capability of being seamlessly incorporated into inexpensive and mobile detection apparatus [92,93,94], rendering them appropriate for conducting on-site analyses within the food sector.

Nevertheless, the consumption of quantum dots (QDs) in food-related contexts also elicits apprehension regarding their safety and possible toxicity [95]. Ensuring the non-toxicity and biocompatibility of quantum dots utilised in food detection applications is of utmost significance [96]. Ongoing research is being conducted to evaluate the safety of quantum dots (QDs) and to devise strategies for the secure disposal of QDs and materials containing them.

## 10. Food Packaging Applications of QD–Polymer Nanocomposites for Food Safety

Ensuring the safety of food is a matter of utmost importance for various stakeholders, including consumers, manufacturers, and regulatory agencies [97]. The role of packaging in safeguarding the safety and quality of food is crucial, as it serves to shield it from potential physical, chemical, and biological hazards. The employment of nanotechnology in the development of advanced food packaging materials with improved functionality has garnered growing attention in recent times [98]. QD–polymer nanocomposites are a type of material that exhibit distinctive optical and electronic characteristics, rendering them appropriate for a range of food packaging purposes [4].

The utilisation of QD–polymer nanocomposites in food packaging is a significant area of application, particularly in the detection of microbial contamination and spoilage [99]. Quantum dots (QDs) have the potential to be integrated into the polymer matrix of food packaging materials [100]. Upon exposure to contaminants, these QDs emit a fluorescence signal that can be detected by a fluorescence detector. The aforementioned process facilitates the prompt and precise identification of spoilage and contamination, thereby enabling timely intervention to impede the proliferation of deleterious microorganisms [28].

Quantum dots (QDs) have the potential to serve as a means of temperature monitoring for food products throughout their storage and transportation processes [96]. QD–polymer nanocomposites have the potential to exhibit a chromatic alteration or fluorescence emission at a predetermined temperature, thereby serving as a visual indicator of temperature fluctuations [96]. This is especially advantageous for comestibles that are sensitive to temperature and necessitate precise temperature regulation to preserve their quality and safety [101].

The incorporation of QD–polymer nanocomposites has been shown to enhance the oxygen and moisture barrier characteristics of food packaging materials [102]. Quantum dots (QDs) have the potential to serve as additives in the polymer matrix, thereby improving the barrier characteristics of the packaging material [103]. This can lead to a reduction in the rate of transmission of oxygen and moisture through the packaging material. Food preservation techniques aid in maintaining the freshness and quality of food, thereby prolonging the shelf life of perishable food items [104]. QD–polymer nanocomposites exhibit antimicrobial characteristics that can impede the proliferation of deleterious microorganisms on the packaging material’s surface. The aforementioned outcome is attained through the integration of antimicrobial agents into the polymer matrix, subsequently activated by the fluorescence emitted by the QDs [105]. The implementation of this measure may potentially mitigate the incidence of foodborne diseases and enhance the safety standards of packaged food items.

Table 2 depicts the constraints and requirements for food packaging materials. The implementation of QD–polymer nanocomposites in food packaging materials can potentially improve the barrier properties of said materials, leading to an extension of the shelf life of perishable food items. This can have a positive impact on reducing food waste and enhancing the overall quality of the food. The aforementioned objective is attained by mitigating the pace of oxygen and moisture permeation via the packaging medium, as these factors are known to instigate food spoilage and oxidation [106]. The utilisation of QD–polymer nanocomposites has the potential to enhance the safety of packaged foods through the detection of hazardous microorganisms and the inhibition of their proliferation on the packaging material’s surface. The implementation of this measure may potentially mitigate the incidence of foodborne disease and enhance the trust of consumers in the safety of pre-packaged food products [107]. QD–polymer nanocomposites have the potential to enhance sustainability efforts by prolonging the shelf life of food and minimising food waste. Furthermore, quantum dots (QDs) exhibit non-toxic and biocompatible properties, rendering them a secure option for employment in food packaging materials. The production expenses associated with QD–polymer nanocomposites may exceed those of conventional food packaging materials [108]. As ongoing research and development in this domain persists, it is probable that the expense will diminish gradually. Despite the non-toxic and biocompatible nature of QDs, apprehensions regarding their safety in food packaging materials persist [109]. Further investigation is required to comprehensively comprehend the plausible health and safety hazards linked with QD–polymer nanocomposites. Regulatory approval may be necessary in certain regions for the implementation of QD–polymer nanocomposites in food packaging materials [110]. Adherence to pertinent regulations and standards is crucial in guaranteeing the safety of consumers when utilising these materials.

## 11. Monitoring and Sensing Applications of QD–Polymer Nanocomposites

In the world of food packaging, QD–polymer nanocomposites are emerging as a viable platform for a variety of monitoring and sensing applications [121]. These nanocomposites can be designed to have special optical and electrical characteristics that can be used to track a variety of food packaging-related factors, including temperature, gas permeability, moisture content, and freshness [122]. Oxygen detection is one of the main uses of QD–polymer nanocomposites in food packaging. Oxygen-sensitive QDs can be used to evaluate the freshness of food goods since oxygen causes them to modify their fluorescence properties [123]. To make sure that the food is stored at the proper temperature, temperature-sensitive QDs can also be employed to monitor temperature changes in food packaging. Antimicrobial capabilities are another use for QD–polymer nanocomposites in food packaging [124]. To release antimicrobial drugs in response to certain stimuli, such as temperature or pH changes, or in the presence of particular microbes, QDs can be integrated into the polymer matrix. By stopping the growth of dangerous bacteria and other germs, this can help to extend the shelf life of food products. Another use for QD–polymer nanocomposites is the development of gas barrier coatings for food packaging. The QDs can be integrated into the polymer matrix to produce a homogeneous, dense covering that resists gas permeation, preserving the food’s freshness [98]. Additionally, by identifying the presence of specific volatile organic compounds produced by food as it starts to spoil, QD–polymer nanocomposites can be used to monitor the quality of food during storage and transport. Packaging for food products that is traceable and tamper-evident can also be made using QD–polymer nanocomposites [125]. Incorporating QDs into the polymer matrix can produce a distinctive fluorescence or colour pattern that is challenging to duplicate, assisting in the prevention of tampering and ensuring the integrity and safety of the food product [97,126,127]. Additionally, QDs can be encoded with distinctive data that can be read by a fluorescence or spectral analysis system, such as the origin of the food product or its expiration date. By preventing counterfeiting, this can aid in supply chain management improvement.

**Figure 6:**
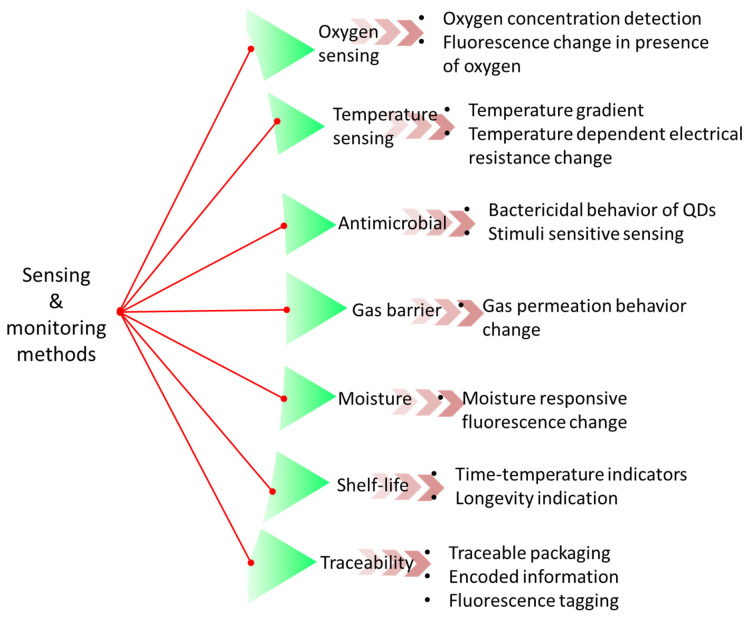
Various modes of sensing and monitoring paths to detect adulteration and food spoilage by using QD–polymer nanocomposites.

The antibacterial properties, antioxidant activity, and high compatibility with most biopolymers of QDs make it a promising candidate for the development of active food packaging films as functional fillers. Additionally, its low toxicity and water solubility further enhance its suitability for this application. The incorporation of colloidal quantum dots into composite films results in the enhancement of various physicochemical properties, including mechanical strength, UV blocking ability, and gas barrier properties. Table 3 depicts various types of QD-reinforced polymer composite films that have been applied in food packaging. This is attributed to the formation of robust interactions between the QDs and the polymer matrix, facilitated by hydrophilic interactions. Additionally, the resulting composite films exhibit both antioxidant and antibacterial properties. Table 3 presents a comprehensive overview of the impact of incorporating QDs on the physical, chemical, and functional characteristics of biodegradable food packaging films. There are several ways to detect food spoilage and monitoring to evaluate adulteration qualitatively as well as quantitatively (Figure 6). To date, two primary techniques have been employed for the production of active films based on antimicrobial QDs, namely, absorption/coating and solvent casting through direct incorporation. The study involved the creation of Enoki mushroom-derived carbon dots (mCDs) through a straightforward hydrothermal process. These mCDs were then utilised in the production of active food packaging films made from gelatine and carrageenan [128]. The samples exhibited significant antioxidant properties while exhibiting minimal toxicity towards mouse fibroblast L929 cells. Additionally, they were uniformly distributed within the gelatine/carrageenan polymer matrix, resulting in the formation of a compatible composite film. The incorporation of QDs resulted in the formation of a film that exhibited enhanced mechanical properties and high transparency, while maintaining the water vapour permeability and hydrophobicity of the original film.

**Figure 7:**
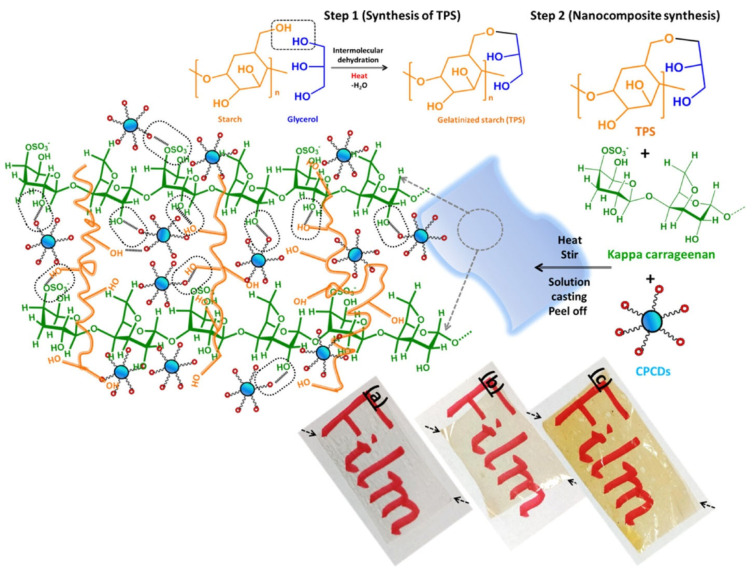
Diagrammatic representation of the synthesis of TPS and the formation of TPS-κ-carrageenan film based on CPCD. The aforementioned figures depict digital representations of the unloaded film TC0 (**a**), as well as the CPCD-loaded hybrid films TC1 (**b**) and TC2 (**c**). Reproduced with permission from Ref. [4]. 2022 American Chemical Society.

Polymeric thin films that are flexible, antioxidant, and UV-resistant have been produced using a simple physical compounding technique, with reinforcement from heteroatom-doped carbon dots (CDs) (Figure 7) [4]. The optical characteristics, photostability, and radical-scavenging activities of the CDs were found to be satisfactory. The study assessed the capacity of CDs to scavenge free radicals through the use of DPPH, hydroxyl (OH) radical, and ABTS radical cation decolourisation assays. Furthermore, the hybrid films exhibited an enhanced polarity-based sustained-release mechanism for the encapsulated active agents, namely, CPCDs, across diverse simulated food environments. According to the release kinetics, it was observed that over 60% of the CPCD could be released within the first two hours. The evaluation of the release mechanism of CDs from hybrid films was conducted by plotting the initial plateaued data into non-Fickian diffusion models.

## 12. Sensing of Physical and Chemical Parameters in Food Using QD–Polymer Nanocomposites, such as Temperature, Humidity, pH, and Gases

The utilisation of quantum dot–polymer nanocomposites in detecting physical and chemical parameters in food presents notable benefits, especially in the context of food packaging. The integration of sensors into food packaging is a crucial element in ensuring the preservation and safety of food. This approach enables the real-time monitoring of diverse parameters that impact the quality and safety of food [143]. The utilisation of QD–polymer nanocomposites for temperature sensing purposes can be integrated into food packaging materials with the aim of monitoring the temperature of the food product [4]. The sensors have the capability to alter their chromatic properties or radiate light upon the attainment of a predetermined temperature threshold, thereby signifying the exposure of food to unsuitable thermal conditions [144]. These data can be utilised to guarantee that the food item stays within acceptable temperature thresholds while being transported, stored, and distributed [145]. The integration of QD–polymer nanocomposites for humidity sensing purposes can be extended to the realm of food packaging materials. The sensors possess the capability to perceive alterations in humidity levels and furnish data concerning the moisture composition of the food item [146]. Elevated levels of humidity have the potential to induce food spoilage, whereas decreased levels of humidity can lead to desiccation and the degradation of the food’s sensory attributes [147]. Through the monitoring of humidity levels, adjustments can be made to the packaging material in order to sustain the ideal humidity conditions for the food product. The utilisation of QD–polymer nanocomposites for pH sensing purposes can be integrated into food packaging materials to effectively track variations in the acidity or alkalinity levels of the food item [148]. The sensors possess the capability to perceive alterations in pH levels and furnish data pertaining to the degree of freshness of the comestible item [149]. Variations in pH levels may serve as an indicator of the proliferation of deleterious microorganisms, which may lead to food spoilage and jeopardise human well-being [150]. The integration of QD–polymer nanocomposites for gas sensing purposes can be extended to the domain of food packaging materials. The sensors possess the ability to perceive alterations in gas concentrations, specifically oxygen (O_2_), carbon dioxide (CO_2_), and nitrogen (N_2_). This capability can furnish insights regarding the standard and recentness of the comestible item. Through the monitoring of gas concentrations, it is possible to make adjustments to the packaging material in order to sustain the ideal conditions for the food product [151]. The utilisation of QD–polymer nanocomposites in the context of food packaging has the capacity to enhance the safety and quality of food products through the provision of the instantaneous monitoring of both physical and chemical parameters [152]. The incorporation of sensors into food packaging materials has the potential to furnish significant insights regarding the quality and safety of food items. This can facilitate improved decision making with respect to storage, transportation, and distribution.

## 13. Detection and Quantification of Food Quality Parameters Using QD–Polymer Nanocomposites, Such as Freshness, Ripeness, and Flavour

The safety and satisfaction of consumers depend heavily on the quality of the food. Food quality parameters including freshness, ripeness, and flavour are crucial signposts that can affect the sensory experience of eating [153]. Traditional methods for evaluating food quality can take a long time, cost a lot of money, and call for specialised tools and qualified employees. Therefore, the need for the creation of quick, accurate, and economical methods for evaluating food quality parameters is increasing [154]. QD-incorporated polymer nanocomposites have become a viable method for identifying and measuring food quality indicators. By adding QDs to a polymer matrix, these nanocomposites can be created, producing materials with enhanced mechanical, optical, and electrical capabilities. The selective detection and quantification of target analytes may be made possible by functionalising QDs with particular biomolecules or polymers [155]. QDs can be functionalised with biomolecules that bind to the volatile organic compounds (VOCs) produced by spoiled food in order to detect freshness. For instance, antibodies or aptamers that bind to particular VOCs linked to food rotting can be used to functionalise QDs. The amount of freshness may be measured thanks to the VOCs’ binding to the QDs, which causes a drop in their fluorescence intensity. Similar to this, QDs can be functionalised with antibodies or aptamers that specifically attach to proteins linked to ripeness in order to detect its presence. For instance, antibodies that bind to particular ethylene receptors linked to fruit ripening can be used to functionalise QDs [156,157,158,159,160]. The binding of the ethylene receptors to the QDs causes a shift in the fluorescence intensity or spectrum, which allows the level of ripeness to be measured as the fruit ripens and the concentration of ethylene receptors rises. By integrating QDs into a polymer matrix with a certain porosity or surface chemistry that permits the adsorption of flavour molecules, flavour detection with QDs can be accomplished. For instance, QDs may be added to a porous polymer matrix with a particular surface chemistry that permits the adsorption of particular flavouring substances. Specific flavour compounds can be identified and measured because the QDs can quench the fluorescence of the flavour compounds as they bind to them. Generally speaking, QD–polymer nanocomposites present a promising method for the identification and measurement of food quality indicators. To maximise the sensitivity and specificity of these systems for various food types and quality characteristics, a number of issues must be resolved. These difficulties include designing and choosing appropriate biomolecules or polymers for QD functionalisation, optimising QD loading and distribution in the matrix of the polymer, and creating solid and dependable techniques for sample preparation and analysis.

## 14. Challenges and Future Perspectives

In spite of the remarkable attributes and significant progressions in the exploration of synthesis techniques and composites of quantum dots and polymers in contemporary times, there exist certain hindrances that necessitate resolution in the future. A viable technique for the production of QD–polymer composites involves physical blending, albeit this method is not without its drawbacks. The non-uniform distribution of quantum dots (QDs) within the polymer matrix presents a notable obstacle [161]. Although in situ polymerisation has the potential to address several drawbacks, there is still room for further enhancements. Another problem is the lack of techniques for the characterisation of QDs during their in situ polymerisation growth [162]. A significant constraint in the utilisation of QDs is their potential toxicity due to the presence of heavy metals used in their production, which can pose a risk to human health. Despite attempts to address the problem, such as the creation of QDs using less harmful substances or the application of biocompatible coatings, the issue remains a noteworthy area of concern. In addition, the utilisation of QDs in the domains of food safety and sensing necessitates careful regulation of their characteristics, encompassing dimensions, morphology, and surface composition [163]. Attaining such a degree of control may turn out to be a difficult task, necessitating the utilisation of specialised apparatus and a high level of proficiency [164]. In addition, the expense involved in the fabrication of QDs and integrating them into food safety and sensing systems is a factor that prevents their broad application. Various prospective approaches and tactics exist for overcoming the obstacles related to the utilisation of QD–polymer nanocomposites in the domains of food safety, monitoring, and sensing. Those include (i) developing heavy-metal-free QDs, (ii) surface functionalisation and coating of QDs to improve their compatibility with food matrices and enhance their sensing properties, and (iii) cost reduction. In brief, in order to overcome the challenges that are associated with the application of QD–polymer nanocomposites in food safety, monitoring, and sensing applications, a multidisciplinary approach is required. This approach involves the development of QDs that are safer and more compatible, the improvement of manufacturing and characterisation methods, and the reduction of costs through increased efficiency and scalability [165]. In future, additional investigation and advancement in this area have the potential to yield innovative and enhanced sensing platforms for the purpose of monitoring food safety. This can facilitate the rapid and precise identification of contaminants and spoilage, while also minimising food waste and enhancing public health. The integration of quantum dots and polymers has the capacity to generate novel market opportunities and stimulate economic expansion. These composite materials have diverse applications across multiple industries, including, but not limited to, electronics, energy, and healthcare. Quantum dot–polymer composites have potential applications in various fields such as high-resolution displays, advanced sensors, efficient solar cells, and biomedical imaging devices. The proliferation of these applications can potentially lead to a rise in economic activity and the generation of employment opportunities. The incorporation of quantum dots has the potential to augment the performance attributes of polymers. These materials demonstrate distinctive optical characteristics, including adjustable absorption and emission spectra, elevated quantum yields, and limited emission linewidths. The integration of quantum dots within polymer matrices can lead to composites that demonstrate heightened electrical conductivity, superior light absorption and emission, and effective energy transfer. The optimisation of performance has the potential to facilitate the emergence of novel products that exhibit enhanced functionality, thereby conferring a competitive edge to enterprises and stimulating market demand.

## 15. Conclusions

In summary, the optical and electronic properties of quantum dot–polymer nanocomposites have rendered them highly promising for employment in the domains of food safety, monitoring, and sensing. Noteworthy discoveries and contributions have been documented in these studies. Numerous investigations have been conducted regarding the prospective uses of QD–polymer nanocomposites in this domain with key findings and contributions including improved sensitivity and selectivity, better reliability and compatibility, swift monitoring, expedited screening, and economical and expandable manufacturing. Food contamination and spoiling can be detected quickly and accurately, lowering the risk of foodborne illness and improving public health. Furthermore, using QD–polymer nanocomposites in food quality monitoring may decrease food waste and increase food shelf life, thus improving consumers’ as well as manufacturers’ satisfaction.

## Figures and Tables

**Table 1:** Different types of food items and their commercially viable spoilage detection techniques.

Food Application	Detection Method	Advantages	Limitations	Ref.
Meat	Fluorescence	High sensitivity, selectivity, and stability; rapid detection time	Expensive synthesis, toxicity concerns	[70]
Milk	Colorimetric	Low cost, simple detection	Limited selectivity	[71]
Seafood	Fluorescence	High sensitivity, selectivity, and stability; rapid detection time	Expensive synthesis, toxicity concerns	[72]
Fruits	Surface-enhanced Raman scattering (SERS)	High sensitivity and selectivity; non-destructive detection	Limited stability	[73]
Fruit juice	SERS	High sensitivity and selectivity; non-destructive detection	Expensive synthesis	[74]
Vegetables	Electrochemical	High sensitivity and selectivity; rapid detection time	Limited specificity, requirement for trained personnel	[75]
Cheese	Fluorescence	High sensitivity and selectivity; rapid detection time	Limited stability, interference from cheese matrix	[76]
Bread	Colorimetric	Low cost, simple detection; real-time monitoring	Limited sensitivity	[77]
Wine	SERS	High sensitivity and selectivity; non-destructive detection	Expensive synthesis, limited stability	[78]
Yogurt	Fluorescence	High sensitivity, selectivity, and stability; rapid detection time	Interference from yogurt matrix	[79]

**Table 2:** Different types of constraints affecting food packaging systems and properties.

Food Packaging			
Constraints	Significant Features	Materials Used	Ref.
Active packaging	QD–polymer nanocomposites can be utilised as active packaging to prevent food spoiling by releasing antibacterial agents or antioxidants. pH, temperature, and moisture variations can release these chemicals.	Carboxymethyl cellulose (CMC) and CQDs	[111]
Sensor integration	QD–polymer nanocomposites can be used in food packaging to monitor freshness, ripeness, and contamination. These sensors can respond to oxygen, carbon dioxide, and volatile organic molecules by changing colour, fluorescence, or electrical conductivity.	Poly(acrylic acid), poly(2-vinylpyridine), GQDs	[112]
Tamper-evident packaging	QD–polymer nanocomposites can be used in food packaging to monitor freshness, ripeness, and contamination. These sensors can respond to oxygen, carbon dioxide, and volatile organic molecules by changing colour, fluorescence, or electrical conductivity.	Pyrene-based chalcone	[113]
Environmental monitoring	QD–polymer nanocomposites can monitor temperature, humidity, and pH to ensure food safety. QDs embedded in food packaging polymer matrices may detect environmental conditions by changing fluorescence.	Polyarylene ether nitrile, ZnCdSe/ZnS QD	[114]
Traceability and Authentication	QD–polymer nanocomposites can create food packaging with unique identification codes or patterns for traceability and authenticity. Scanners or cell phones can read these codes or patterns to authenticate the packed food’s identity and origin.	Epoxy InP@ZnS QDs	[115]
Environmental Monitoring	QD–polymer nanocomposites can monitor temperature, humidity, and pH to ensure food safety. QDs embedded in food packaging polymer matrices may detect environmental conditions by changing fluorescence.	Molecularly imprinted polymer, GQDs	[116]
Gas Barrier Properties	QD–polymer nanocomposites can minimise oxygen and water vapour permeability in food packaging. This helps preserve packaged foods.	PMMA, ZnS/ZnS QDs	[100]
Nanoparticle Migration	QD–polymer nanocomposites may leak nanoparticles into food. Since some nanoparticles may impair human health, this is crucial for assessing food packaging safety.	Polyethylene, CdSe QDs	[117]
Biodegradability	QD–polymer nanocomposites made from biodegradable polymers lessen environmental effect. Sustainable food packaging reduces waste and pollution.	Poly(lactide), Organic Quantum Dots (Qdot^®^655 ITK™; catalogue number 2172-1)	[118]
Regulatory Considerations	The FDA regulates QD–polymer nanocomposites. QD-containing food packaging must be safe and tested.	Poly [2-methoxy-5-(2′-ethylhexyloxy-p-phenylenevinylene)], PbS QDs	[119]
Cost Considerations	QD–polymer nanocomposites in food packaging pricing can affect their economic viability. Cost-effective manufacturing processes and production scale are needed to use these materials in food packaging.	Poly(2-methoxy-5-(2-ethylhexyloxy)-1,4phenylenevinylene), GQDs	[120]

**Table 3:** Various types of QDs based polymer composites and their end usage in food packaging.

Matrix Phase	QDs	Composite Special Feature	Application Area	Ref.
Nanocellulose	4,7,10-trioxa-1,13-tridecanediamine	UV resistance, thermal stability	UV-protective packaging	[129]
PVA	Citric acid and imine	UV barrier	UV-protective packaging	[130]
PMMA	Carbon black	UV barrier	UV-protective packaging	[131]
Regenerated cellulose	Lactose	High fluorescence	-	[132]
Collagen	Cotton	UV resistance	Biodegradable UV-protective packaging	[133]
PVA	Residue of *Radiata pine*	Anti-counterfeiting	Anti-fake packaging	[134]
Chitosan	Kelp	Bactericidal	Cucumber storage and packaging	[135]
PVA	Tea residue	UV protection	Grape packaging	[136]
Chitosan	Banana	Bactericidal	Soy milk packaging for high shelf life	[137]
PVA	Cyanobacteria	IV/IR barrier, anti-counterfeiting	Anti-fake packaging	[138]
Polypyrrole/chitosan blend	Citric acid and amino acid	Bactericidal	Antibacterial food packaging	[139]
BC	Postbiotics of *L. acidophilus*	Bactericidal	Antimicrobial packaging	[140]
Zein	Zinc acetate	Antibacterial	Antimicrobial packaging	[141]
Gelatine/carrageenan	Enoki mushroom	Antioxidant, UV barrier, high mechanical, antibacterial	Antioxidant and antibacterial packaging	[128]
Pectin/gelatine	Turmeric	Antibacterial	Antibacterial packaging	[142]
TPS/carrageenan	Clove	Antioxidant, bactericidal	Antibacterial and antioxidant packaging	[4]

## Data Availability

The data are available from the corresponding author.
